# Chromobox Homolog 8 (CBX8) in Human Tumor Carcinogenesis and Prognosis: A Pancancer Analysis Using Multiple Databases

**DOI:** 10.3389/fgene.2021.745277

**Published:** 2021-09-09

**Authors:** Dongjie Shi, Lei Ao, Hua Yu, Yongzhi Xia, Juan Li, Wenjie Zhong, Haijian Xia

**Affiliations:** ^1^Department of Neurosurgery, The First Affiliated Hospital of Chongqing Medical University, Chongqing, China; ^2^Clinical Skill Training Center, The First Affiliated Hospital of Chongqing Medical University, Chongqing, China

**Keywords:** pancancer analysis, prognosis, carcinogenesis, cancer, CBX8

## Abstract

Some emerging studies have suggested that chromobox homolog 8 (CBX8) may play a critical role in carcinogenesis and prognosis in human cancer. Based on The Cancer Genome Atlas (TCGA)’s available data and the Gene Expression Omnibus (GEO) database, we conducted a systematic analysis of the carcinogenic effects of the CBX8 gene. We used TIMER2, GEPIA2, UALCAN, cBioPortal, Kaplan-Meier plotter, OncoLnc, STRING, HPA, and Oncomine data analysis websites and R data analysis software to analyze available data. The results show that the level of expression of CBX8 was significantly different among 27 different types of tumors and adjacent normal tissues. Moreover, we found that CBX8 expression had a close relationship with prognosis in some kinds of cancers. The phosphorylation level of some protein sites (such as S256) was significantly increased in tumors. CD8 + T-cell, B-cell and cancer-associated fibroblast infiltration levels were associated with CBX8 expression. The results of enrichment analysis indicated that the main biological activities of CBX8 are connected to gene transcription and repair of DNA damage. In conclusion, the level of expression of CBX8 was closely related to carcinogenesis and prognosis of some kinds of tumors, which needs further experimental verification.

## Introduction

Chromobox homolog 8 (CBX8), also called Human Polycomb 3, is an important member of Polycomb Group (PcG) proteins, which were first reported as critical developmental regulators in Drosophila (Benetatos et al., 2014; Chiacchiera et al., 2016). As an important factor involved in cell life activities, CBX8 inhibits the transcription of some critical target genes, including the INK4a/ARF gene (a tumor-suppressing gene affecting the survival of cells) (Dietrich et al., 2007) and the AF9 gene (related to acute leukemia occurrence) (Tan et al., 2011), and can regulate cell differentiation (Tang B. et al., 2019). In recent years, a link between CBX8 and tumors has been consistently found.

According to some published studies, in hepatocellular carcinoma, the proliferation, migration and invasion of hepatocellular carcinoma are affected by CBX8. Moreover, the overexpression of CBX8 indicates a poor prognosis (Tang B. et al., 2019). A recent study indicated that high expression of CBX8 indicates a poor prognosis in breast cancer. This finding may be related to the promotion of Notch signal expression by CBX8 (Chung et al., 2016). The transcriptional regulation of mixed lineage leukemia (MLL)-AF9 and the occurrence of leukemia are affected by CBX8 (Tan et al., 2011). However, there is no pancancer analysis to comprehensively assess the relationship between CBX8 expression and carcinogenesis and prognosis for various cancers.

Based on functional genomics datasets of different tumors from the TCGA project and GEO database (Clough and Barrett, 2016; Blum et al., 2018), we first systematically evaluated the association between CBX8 and tumors. We performed CBX8 expression, clinical prognosis, gene alteration, protein phosphorylation, tumor microenvironment immune infiltration and gene enrichment analyses to explore the potential role of CBX8 in the pathogenesis or clinical prognosis of different cancers.

## Materials and Methods

### Tumor Immune Estimation Resource, Version 2 (TIMER2) (web: http://timer.cistrome.org/) (Li et al., 2016, 2017, 2020)

We put CBX8 into the “Gene_DE” module of the “Exploration” section and observed the differences in the expression of CBX8 between adjacent normal tissues and different tumors or specific tumor subtypes of the TCGA project.

We used the “Gene” module of the “Immune” section to explore the association between CBX8 expression and immune infiltrates of all available tumors in the TCGA project. The selected immune cells were CD8 + T cells, B cells, and cancer-associated fibroblasts. Immune infiltration was estimated by part or in all of the TIMER, CIBERSORT, CIBERSORT-ABS, QUANTISEQ, XCELL, MCPCOUNTER, and EPIC algorithms. The *P*-values were obtained by purity-adjusted Spearman’s rank correlation tests. We used heatmaps and scatter plots to visualize the data.

The “Gene_Corr” module of the “Exploration” section was used to create the heatmap of the selected genes, which contains the partial correlations (cor) and *P*-values (calculated by purity-adjusted Spearman’s rank correlation tests).

### Gene Expression Profiling Interactive Analysis, Version 2 (GEPIA2) (web: http://gepia2.cancer-pku.cn/#analysis) (Tang Z. et al., 2019)

For certain tumors without correlated normal tissues (e.g., TCGA-DLBC, TCGA-UCS) in the TCGA project, we obtained the corresponding normal tissues in the Genotype-Tissue Expression (GTEx) database and used the “Expression Analysis-Box Plots” module to obtain box plots of the expression differences between these tumor tissues and the normal tissues (settings: *P*-value Cutoff = 0.01, Ilog2FCl Cutoff = 1, and “Match TCGA normal and GTEx data”).

We obtained violin plots of CBX8 expression in different pathological stages (stage I, stage II, stage III, and stage IV) of all TCGA tumors with the “Pathological Stage Plot” module of GEPIA2. The log2 [transcripts per million (TPM) + 1] transformed expression data were applied for the box or violin plots.

The “Survival Analysis” module was used to evaluate the significant relationship between overall survival (OS) and disease-free survival (DFS) and CBX8 expression for all tumors in the TCGA project (settings: cutoff-high: 50%, cutoff-low: 50%). The hypothesis tests used log-rank tests. These data were visualized by survival plots.

In the TCGA project, we obtained the top 100 target genes correlated with CBX8 via the “Similar Gene Detection” module and used pairwise Pearson correlation analysis to analyze the correlation between CBX8 and these target genes. For the two highest correlation coefficients (R), we used a dot plot to visualize the data.

### UALCAN (web: http://ualcan.path.uab.edu/analysis-prot.html) (Chandrashekar et al., 2017)

We used the Clinical Proteomic Tumor Analysis Consortium (CPTAC) dataset to analyze the expression levels of total protein and phosphoprotein between six kinds of tumors and adjacent normal tissues. The available datasets of tumors were selected, including breast cancer, clear cell renal cell carcinoma (RCC), colon cancer, lung adenocarcinoma (LUAD), ovarian cancer, and uterine corpus endometrial carcinoma (UCEC). The enrolled phosphorylation sites included S110, S191, S195, S196, S256, T264, S265, S311, and S526.

### cBioPortal (web: https://www.cbioportal.org/) (Cerami et al., 2012; Gao et al., 2013)

We chose the “TCGA Pan Cancer Atlas Studies” in the “Query by gene” section and included “CBX8” to obtain the mutation characteristics of CBX8. In the “Cancer Types Summary” module, we observed the results of the alteration frequency, mutation type and copy number alteration (CNA) of all tumors in the TCGA project.

In the “Comparison” module, we obtained data on the OS, DFS, progression-free survival PFS, and DFS differences between those with CBX8 genetic alterations and those without CBX8 genetic alteration for TCGA cancer cases. Kaplan-Meier plots were used to visualize the data.

### Kaplan-Meier Plotter (web: https://kmplot.com/analysis/) (Gyorffy et al., 2010; Menyhart et al., 2018; Nagy et al., 2021)

After choosing different kinds of tumors, including breast cancer, liver hepatocellular carcinoma (LIHC), kidney renal clear cell carcinoma (KIRC), and pancreatic adenocarcinoma (PAAD), we entered CBX8 (settings: Auto select best cutoff, user selected probe set) into the “Affy id/gene symbol” module and obtained prognosis data for tumor patients, which were visualized by Kaplan-Meier plots.

### OncoLnc (web: http://www.oncolnc.org/) (Anaya, 2016)

Chromobox homolog 8 was entered in the search column, and the relevant *P*-values and Cox coefficients were obtained based on Cox regression analysis of the prognostic data of patients with 21 kinds of tumors in the TCGA project.

### STRING (web: https://string-db.org/) (Szklarczyk et al., 2019)

In the “Protein by name” module, we put CBX8 in the query column and set the organism to Homo sapiens. The following settings were used: “Low confidence (0.150),” “evidence,” “no more than 50 interactors,” and “experiments.” Then, we obtained 50 experimentally verified CBX8-binding protein datasets.

### The Human Protein Atlas (HPA) (web: https://www.proteinatlas.org/) (Uhlen et al., 2015, 2017; Thul et al., 2017)

By entering the single gene CBX8 in the search column and choosing the “tissue” module, we obtained the RNA Expression Overview map combined with the HPA, GTEx, and functional annotation of the mammalian genome 5 (FANTOM5) datasets. Then, we chose the “cell type” and “blood” modules and obtained the related RNA Expression Overview maps.

### Oncomine (web: https://www.oncomine.org/)

In Oncomine, we put CBX8 in the search column and chose the “Cancer vs. normal analysis” module for differential analysis. Selecting all relevant data for comparison, we obtained visualized differential expression data between tumors and normal tissues.

### R Software (Version: 4.0.3, 64-bit) (https://www.r-project.org/)

Finally, we combined two sets of data (CBX8-binding and interacting genes) and transformed “hgnc_symbol” into “ensembl_gene_id” with the “biomaRt” R package (Durinck et al., 2005, 2009). In addition, we applied the “clusterProfiler” (Yu et al., 2012) R package to conduct gene ontology (GO) and Kyoto Encyclopedia of Genes and Genomes (KEGG) enrichment analyses. The results of enrichment analysis were visualized by the “ggplot2” (Wilkinson, 2011) and “enrichplot” (Yu et al., 2010) R packages. A *P*-value < 0.05 was considered statistically significant.

### Phylogenetic Tree

We got the CBX8 protein sequences of different biological species. COBALT (Constraint-based Multiple Alignment Tool) was used to match the same and different parts of the CBX8 protein sequence and make the Phylogenetic Tree.

## Results

### Gene Expression Analysis

In our study, phylogenetic tree data (Supplementary Figure 1) were used to determine the evolutionary relationships among the CBX8 proteins of different species. Next, we analyzed the level of CBX8 expression (RNA) in different human tissues. As shown in Supplementary Figure 2A, the results from the combined HPA, GTEx, and FANTOM5 datasets indicated that the highest expression of CBX8 was in the epididymis, followed by granulocytes and fallopian tubes. In all kinds of tissues, CBX8 showed low RNA tissue specificity. For the analysis of CBX8 expression in different single cell types (in all single cell tissues, Supplementary Figure 2B) and different blood cells (using the consensus datasets of the HPA scaled dataset, the Monaco scaled dataset, and the Schmiedel dataset, Supplementary Figure 2C), all results showed low RNA cell specificity.

**FIGURE 1:**
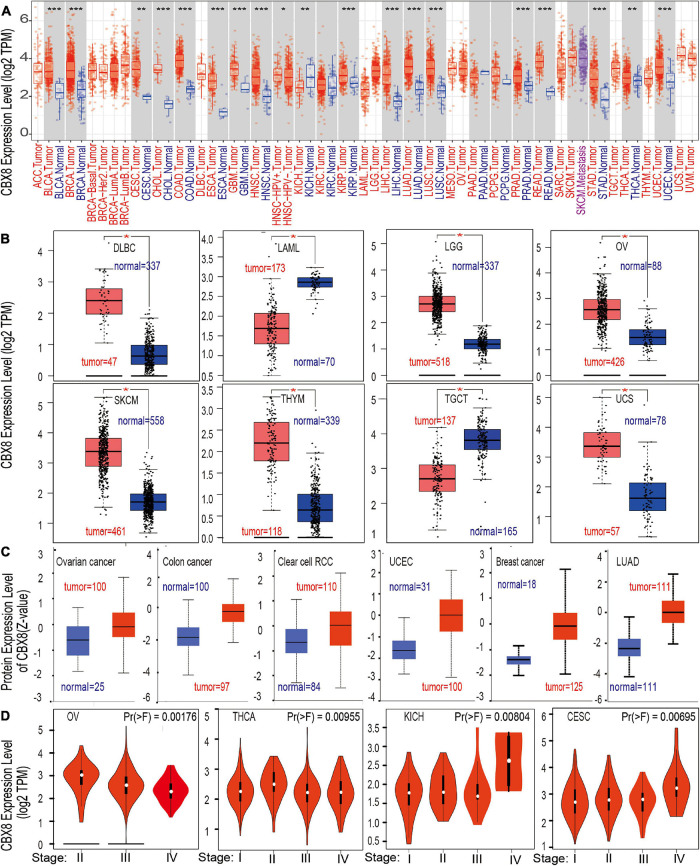
The CBX8 expression level in different tissues. **(A)** Differences in the expression level of the CBX8 gene (RNA) in different cancers or specific cancer subtypes compared with adjacent normal tissues were analyzed by TIMER2. *P < 0.05; **P < 0.01; ***P < 0.001. **(B)** The corresponding normal tissue data for DLBC, LAML, LGG, OV, SKCM, TGCT, THYM, and UCS were provided by the GTEx database as controls to detect CBX8 expression (RNA) level differences, *P < 0.01. **(C)** Differences in the expression levels between normal tissues and primary tissues from breast cancer, colon cancer, clear cell RCC, lung adenocarcinoma, ovarian cancer, and UCEC were compared using the CPTAC dataset, all *P* < 0.001. **(D)** Differences in the expression level of CBX8 (RNA) were analyzed by different pathological stages (stage I, stage II, stage III, and stage IV) of CESC, KICH, OV, and THCA using TCGA data.

To explore the role of CBX8 in the carcinogenesis and development of various cancers, we analyzed the level of CBX8 expression (RNA) in different tumors and adjacent normal tissues. First, we used TIMER2 to analyze the differences in expression of CBX8 in various cancers and corresponding normal tissues. As shown in Figure 1A, the CBX8 expression levels of bladder urothelial carcinoma (BLCA), breast invasive carcinoma (BRCA), cervical and endocervical cancers (CESC), colon adenocarcinoma (COAD), cholangiocarcinoma (CHOL), esophageal carcinoma (ESCA), glioblastoma multiforme (GBM), head and neck squamous cell carcinoma (HNSC), kidney chromophobe (KICH), KIRP, LIHC, LUAD, lung squamous cell carcinoma (LUSC), prostate adenocarcinoma (PRAD), rectum adenocarcinoma (READ), stomach adenocarcinoma (STAD), UCEC, and thyroid carcinoma (THCA) were higher than those of the adjacent normal tissues (all *P*-values < 0.05).

For tumors lacking related normal tissue data, the GTEx dataset was used as a control group for analysis. We further evaluated the differences in the expression of CBX8 between the normal tissues and tumor tissues of uterine carcinoma (UCS), acute myeloid leukemia (LAML), ovarian serous cystadenocarcinoma (OV), lymphoid neoplasm diffuse large B-cell lymphoma (DLBC), brain lower grade glioma (LGG), skin cutaneous melanoma (SKCM), testicular germ cell tumors (TGCT), and thymoma (THYM) (Figure 1B, all *P*-values < 0.01). However, we did not find a significant difference in adrenocortical carcinoma (ACC) or sarcoma (SARC) (Supplementary Figure 3A). Further analysis of the Oncomine database showed that CBX8 was highly expressed in colorectal cancer, leukemia, liver cancer, lung cancer and sarcoma (Supplementary Figure 4, all *P*-values < 0.05) compared with normal controls.

Through analysis of the CPTAC dataset, we found that CBX8 protein had higher expression in breast cancer, clear cell RCC, colon cancer, LUAD, ovarian cancer, and UCEC (Figure 1C, all *P*-value < 0.001) than in related normal tissues. In the exploration of the correlation between CBX8 expression and tumor pathological stages by the “Pathological Stage Plot” module of GEPIA2, some kinds of tumors were observed to be significantly correlated, including CESC, KICH, OV, and THCA (Figure 1D, all *P*-values < 0.05), but others were not (Supplementary Figure 5).

### Survival Analysis

**FIGURE 2:**
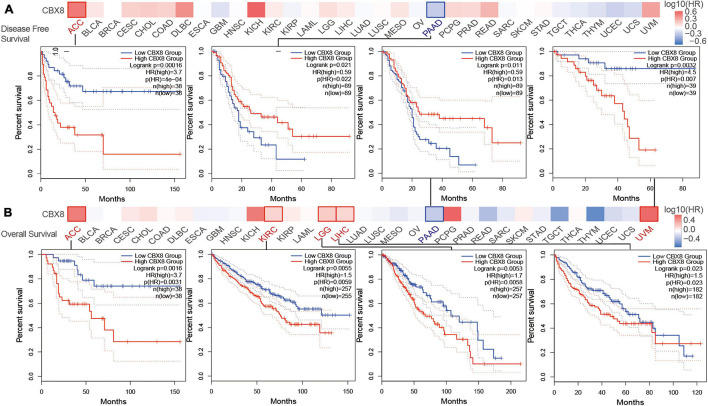
Correlation between CBX8 gene expression and survival prognosis for tumors in TCGA analyzed with the GEPIA2 tool. **(A)** Disease-free survival. **(B)** Overall survival. The results with significant differences were visualized by a survival map and Kaplan-Meier curves.

We divided the included patients into two groups (high-expression group and low-expression group) by the expression of CBX8 (RNA) and compared the two groups on patient survival data. As shown in Figure 2, the high-expression group showed poor OS for uveal melanoma (UVM) (*P*-value = 0.0032), LGG (*P*-value = 0.0053), LIHC (*P*-value = 0.023), KIRC (*P*-value = 0.0055), and ACC (*P*-value = 0.0016) and poor DFS for ACC (*P*-value < 0.001) (Figure 2). In contrast, low CBX8 expression was linked to poor OS (*P*-value = 0.011) and DFS (*P*-value = 0.021) in PAAD.

We used the Kaplan-Meier plotter tool to analyze the survival data. As shown in Supplementary Figure 6A, high CBX8 expression was associated with poor OS (*P*-value = 1e-10) prognosis for KIRC. Additionally, high CBX8 expression had a significant association with poor OS (*P*-value = 0.019), relapse-free survival (RFS, *P*-value = 0.015), and PFS (*P*-value = 0.037) in LIHC (Supplementary Figure 6B). For breast cancer, we observed no significant correlations between CBX8 expression and OS, RFS, post-progression survival (PPS) or distant metastasis-free survival (DMFS) (Supplementary Figure 6C). In contrast, low expression of CBX8 was closely associated with poor OS (*P*-value = 3.9e-5) and RFS (*P*-value = 0.0042) in PAAD (Supplementary Figure 6D). We also conducted subgroup analyses by selected clinical and molecular factors and observed various patterns (Supplementary Table 1).

### Genetic Alteration Analysis

**FIGURE 3:**
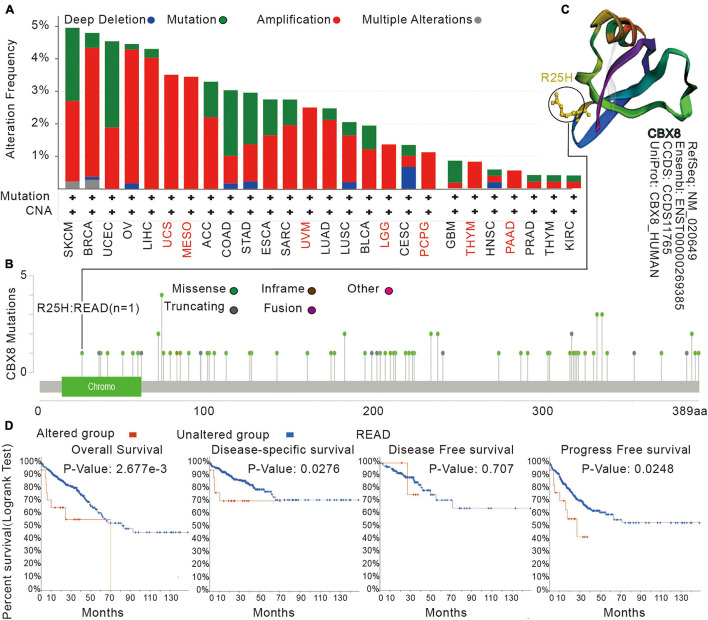
Mutation features of CBX8 in various tumors in TCGA analyzed by the cBioPortal tool. **(A)** The mutation type and alteration frequency in different cancers. **(B)** The mutation sites in CBX8. **(C)** The 3D structure of CBX8 and the mutation site (R25H) in the 3D structure of CBX8. **(D)** The correlation between mutation status and overall, disease-specific, disease-free, and progression-free survival prognoses of READ analyzed by the cBioPortal tool.

By analyzing tumor samples from TCGA cohorts, we obtained the mutation status of CBX8 in different types of tumors. As shown in Figure 3A, amplification was the most common alteration type for CBX8 (RNA) in most kinds of tumors. The “mutation” alteration was the most common type in Uterine Corpus Endometria, which showed an alteration frequency of ∼3%. Amplification was the only CBX8 genetic alteration type for mesothelioma, uterine carcinosarcoma, UVM, LGG, pheochromocytoma and paraganglioma (PCPG), THYM and PAAD. Figure 3B shows the types, sites and case numbers of the CBX8 genetic mutations. Missense mutations were the most common type (62 cases), followed by truncating mutations (14 cases), inframe mutations (1 case) and fusion mutations (1 case). Figure 3C shows the 3D structure of the CBX8 protein. In the R25H alteration, a case of READ was detected, and the site was observed in the CBX8 3D structure. We explored the potential association between genetic alteration of CBX8 and clinical prognosis in different types of cancer. As shown in Figure 3D, READ patients with CBX8 alterations had poorer prognosis for overall survival (*P* = 2.677e-03), disease-specific survival (*P* = 0.0276) and progression-free survival (*P* = 0.0248) than those without CBX8 alterations but were not different in disease-free survival (*P* = 0.707). The association between genetic alteration of CBX8 and clinical prognosis of other cancers are presented in Supplementary Table 2.

### Protein Phosphorylation Analysis

**FIGURE 4:**
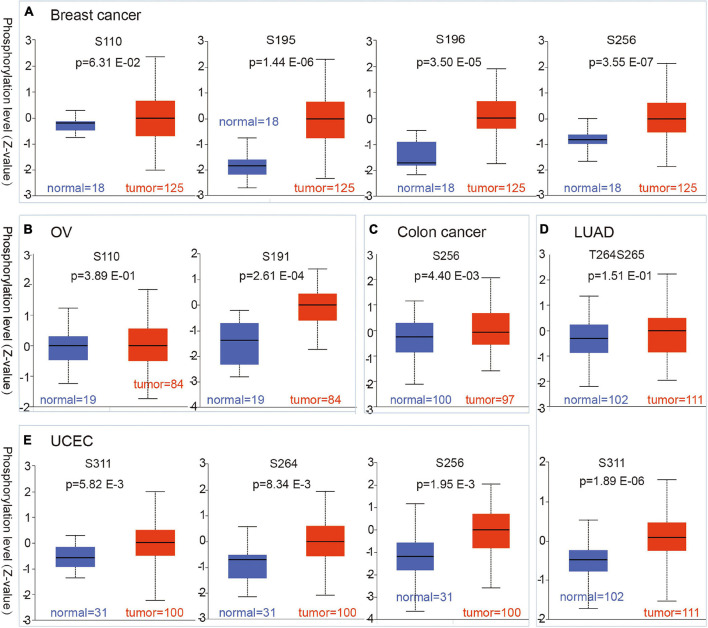
Phosphorylation status of CBX8 protein in different cancers. Based on the CPTAC dataset, the differences in expression of CBX8 phosphoprotein (including phosphorylation sites S191, S195, S196, S256, S264, and S311) between normal tissue and primary tissue from selected tumors are detected via the UALCAN, which was visualized by box plots. **(A)** Breast cancer. **(B)** Ovarian cancer. **(C)** Colon cancer. **(D)** LUAD. **(E)** UCEC.

We used the CPTAC dataset to analyze the CBX8 protein phosphorylation levels of five kinds of tumors (breast cancer, colon cancer, LUAD, ovarian cancer, and UCEC) and compared them with normal tissues. For breast cancer, at sites S256 (*P*-value = 3.55 E-07), S195 (*P*-value = 1.44 E-06), and S196 (*P*-value = 1.50 E-05), CBX8 protein phosphorylation levels were significantly higher than those in adjacent normal tissues, but this association was not observed in S110 (*P*-value = 6.31 E-02) (Figure 4A). Compared with ovarian cancer, adjacent normal tissues had significantly lower phosphorylation levels at S191 (*P* = 2.61 E-04), but not at S110 (*P* = 3.89 E-01) (Figure 4B). The T264 protein site of CBX8 had significantly higher phosphorylation levels in colon cancer than in related normal tissues (*P*-values = 4.40 E-03) (Figure 4C). For LUAD, the difference was significant at phosphorylation site S311 (*P* = 1.89 E-06) but not at phosphorylation sites T264 and S265 (*P* = 1.51 E-01) compared with adjacent normal tissues (Figure 4D). For phosphorylation sites S256 (*P* = 1.95 E-03), T264 (*P* = 1.95 E-03), and S311 (*P* = 5.82 E-03), the phosphorylated levels in UCEC and related normal tissues were significantly different (Figure 4E).

### Immune Infiltration Analysis

**FIGURE 5:**
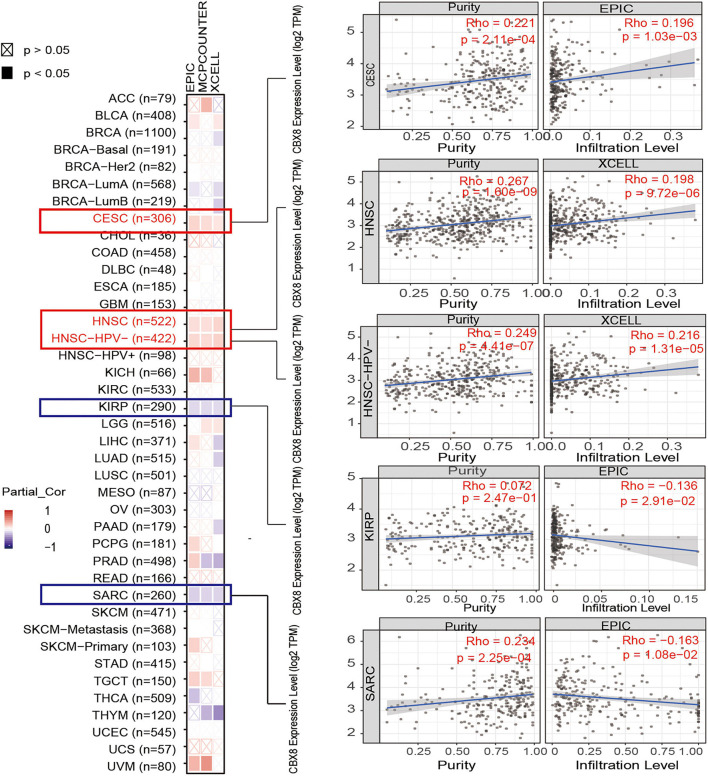
Correlation analysis between CBX8 expression and immune infiltration of cancer-associated fibroblasts. For all types of cancer in TCGA, the correlation between CBX8 expression and immune infiltration of cancer-associated fibroblasts evaluated by different algorithms, including EPIC, MCPCOUNTER, and XCELL.

Tumor-infiltrating immune cells, as an important part of the tumor microenvironment, play critical roles in the initiation, progression, metastasis, and recurrence of tumors (Fridman et al., 2011; Steven and Seliger, 2018). Cancer-associated fibroblasts, CD8 + T cells, and B cells were reported to be closely involved in biochemical processes (Reading et al., 2018; Chen and Song, 2019; El-Daly et al., 2020). We used some or all algorithms (TIMER, CIBERSORT, CIBERSORT-ABS, QUANTISEQ, XCELL, MCPCOUNTER, and EPIC algorithms) to explore the potential association between the infiltration of different immune cells and CBX8 gene expression (RNA) in various tumors. As shown in Figure 5, we used the XCELL, MCPCOUNTER, and EPIC algorithms to investigate the potential association between the infiltration of cancer-associated fibroblasts and the CBX8 gene expression in all kinds of cancers. We observed a statistically negative relationship in KIRP and SARC and a positive association in CESC and HNSC. In the analysis between CD8 + T-cell infiltration and CBX8 expression, the relationship was positive in THYM and UVM and negative in PAAD (Supplementary Figure 7A). For B-cells, the relationship was negative in STAD and THCA but positive in DLBC, LIHC, and PRAD (Supplementary Figure 7B). We used the most statistically significant algorithm to produce scatterplots for all types of tumors. For example, CBX8 expression in KIRP was negatively correlated with infiltration of cancer-associated fibroblasts (cor = −0.136, *P* = 2,910e-2) based on the EPIC algorithm (Figure 5).

### Enrichment Analysis

**FIGURE 6:**
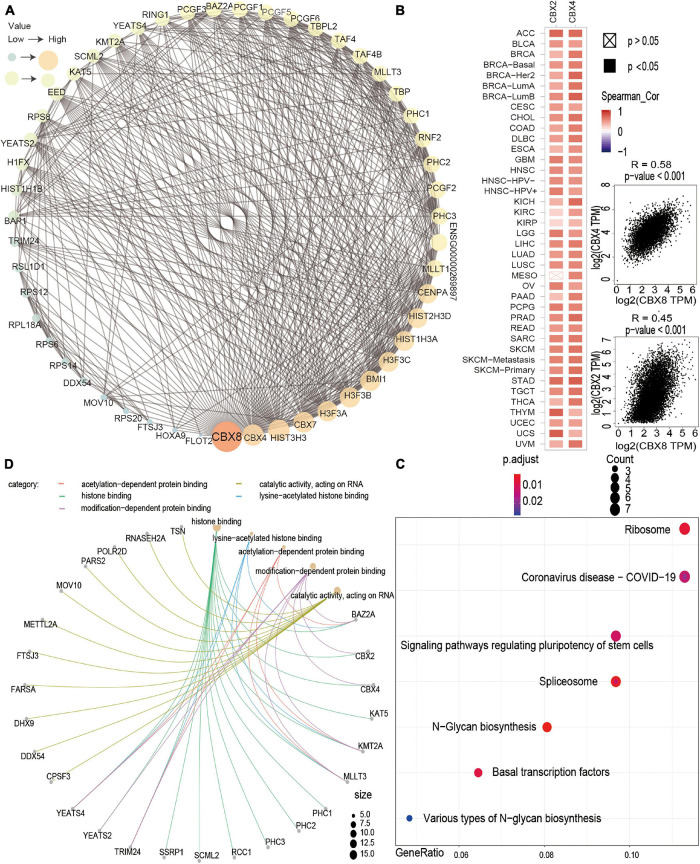
CBX8-related gene enrichment analysis. **(A)** The top 50 CBX8-binding proteins were experimentally determined via the STRING tool and visualized by a molecular interaction network. **(B)** The top 100 CBX8-correlated genes in TCGA projects were obtained via the GEPIA2 tool, and the expression correlation between CBX8 and selected targeting genes, including CBX2 and CBX4, was analyzed. The corresponding heatmap data are displayed for the selected cancer types. **(C)** KEGG pathway analysis based on CBX8-binding and interacting genes. **(D)** GO analysis (molecular function data) based on CBX8-binding and interacting genes.

We investigated the possible molecular mechanism of the CBX8 gene in tumorigenesis by performing pathway enrichment analysis with the CBX8-related gene group, which combines CBX8-binding proteins and CBX8 expression-correlated genes. With the STRING tool, we obtained 50 experimentally verified CBX8-binding proteins. Figure 6A shows the interaction network of these CBX8-binding proteins. We used the GEPIA2 tool to obtain all tumor expression data for TCGA and obtained the top 100 genes related to CBX8 expression. As shown in Figure 6B, we obtained the two genes with the highest correlation with CBX8, including CBX2 (*R* = 0.45) and CBX4 (*R* = 0.58) (all *P*-values < 0.001). The corresponding heatmap data also showed that the two genes have a positive relationship with CBX8 at the expression level in the majority of cancer types (Figure 6B). Next, we combined the two datasets to perform KEGG and GO enrichment analyses. The KEGG results suggest that “spliceosome,” “ribosome,” “basal transcription factors” and “signaling pathways regulating pluripotency of stem cells” might be involved in the effect of CBX8 on tumor pathogenesis (Figure 6C). The GO enrichment analysis results further indicated that most of these gene biological processes are linked to histone modification (including histone ubiquitination, histone H2A monoubiquitination, and others) and covalent chromatin modification (Supplementary Figures 8A,B). The molecular functions included histone binding, catalytic activity acting on RNA, modification-dependent protein binding, acetylation-dependent protein binding, lysine-acetylated histone binding, and others (Figure 6D).

## Discussion

The CBX8 protein is a component of polycomb repressive complex 1 (PRC1), which plays an important role in the cell cycle (Bardos et al., 2000). It has been reported that the main role of CBX8 is regulating cell development, differentiation, and senescence by inhibiting the transcription of downstream genes (Lee et al., 2013) and participating in DNA repair (Tang B. et al., 2019). CBX8 is expressed in a variety of tissues and shows low tissue and cell specificity, which indicates that CBX8 more likely participates in basic activities in cells rather than specific activities. Some emerging studies have shown some potential links between CBX8 and leukemia, hepatocellular carcinoma and breast cancer (Tan et al., 2011; Chung et al., 2016; Tang B. et al., 2019). However, it is undefined whether CBX8 can affect the tumorigenesis, development, and metastasis of different tumors through shared molecular mechanisms. We first analyzed the relationship between CBX8 and various tumors in terms of clinical prognosis and molecular mechanisms using the available TGCT, GEO, and CPTAC datasets.

First, we analyzed differences in the expression of CBX8 in various tumor tissues and corresponding normal tissues. From the relevant results, we can see that there are significant differences in the expression levels of CBX8 between 21 kinds of tumor tissues and corresponding normal tissues. In most kinds of tumors, CBX8 is more highly expressed than in normal tissues. However, the prognostic data for different tumors and CBX8 vary. We further analyzed the relationship between the expression of CBX8 and patient survival with different types of tumors. For LIHC, some studies have shown that high expression of CBX8 induces tumorigenesis and indicates poor prognosis (Zhang et al., 2018; Tang B. et al., 2019). In our study, we analyzed the dataset from the TCGA-LIHC project and found that the overall survival in the high CBX8 expression group was significantly lower than that of the low expression group, which indicates that high expression of CBX8 is correlated with poor overall survival (GEPIA2: *P* < 0.05) in LIHC. At the same time, we used OncoLnc to perform Cox regression for the TCGA-LIHC cohort (Menyhart et al., 2018) and found a significant statistical correlation between CBX8 expression and LIHC patient survival (Cox coefficient = 0.21, *P*-value = 2.50E-03) (Supplementary Table 3). To test these results in different datasets, we used three datasets (RNA-seq, *n* = 371; Affymetrix arrays, *n* = 91; Illumina gene chips, *n* = 135) with the Kaplan-Meier mapping tool to conduct survival analyses. We observed that high CBX8 expression was closely related to poor OS, RFS, and PFS. When we employed some clinical risk factors for subgroup analysis, high expression of CBX8 in the alcohol-consumption group, no hepatitis-virus group, and hepatitis-virus group was still linked to poor prognosis, but the relation was not statistically significant in the no alcohol-consumption group (Supplementary Table 1). Alcohol has been reported to be a risk factor for LIHC patient prognosis, and patients who consume alcohol have a significant reduction in median overall survival (Ganne-Carrie and Nahon, 2019). Thus, additional clinical risk factors should be fully considered in the process to explore the relationship. More molecular experiments are needed to explore whether high CBX8 expression levels play critical roles or only have side effects in the process of cell carcinogenesis.

Emerging evidence shows that high expression of CBX8 is correlated with poor prognosis in breast cancer (Chung et al., 2016). In our study, this relationship was not observed. When we used OncoLnc, the results of Cox regression survival analysis of data in the TCGA-breast cohort also failed to show a statistically significant correlation (Cox coefficient = 0.018, *P* = 0.932) (Supplementary Table 2). Based on the survival data from the Affymetrix HGU133A and HGU133 + 2 microarrays, we observed no significant correlation between CBX8 expression and BRCA patient prognosis (*n* = 4929 cases) (Gyorffy et al., 2010). When we included TP53 for subgroup analysis, high CBX8 expression correlated with good OS in the TP53-mutated group (*n* = 188 cases) and poor RFS in the TP53-wild-type group (*n* = 388 cases) (Supplementary Table 1). The difference in results may be due to the small sample size of the subgroup analysis, which reduced the statistical power of the results (All BRCA patients: *n* = 4,929, TP53-mutated and TP53-wild-type BRCA patients: *n* = 576). It also reminds us that we should combine other molecular biological characteristics for systematic analysis.

For KIRC, we analyzed the TCGA-KIRC cohort with the GEPIA2 tool, and the results showed that patients with high CBX8 expression had poor overall survival. When we used the Kaplan-Meier mapping tool for analysis once again, KIRC patients with low expression of CBX8 had better OS (Nagy et al., 2021). We further included clinical factors for subgroup analysis, and the results showed that high expression of CBX8 correlated with poor prognosis (Supplementary Table 1). In the Cox regression analysis using OncoLnc, we observed that high expression indicated poor patient prognosis (Cox coefficient: 0.355, *P*-value: 8.6E-06) (Supplementary Table 3). However, we did not observe a difference in CBX8 expression between tumor and normal tissues (Figure 1A). Considering the lack of normal tissue samples (tumor: 533 cases, normal: 72 cases), we combined the TCGA and GTEx data as a control group and found that the relationship was still not observed (tumor: 523 cases, normal: 100 cases) (Supplementary Figure 3B). Thus, more normal tissue data for a control group are essential to analyze this relationship and explore whether CBX8 is the initial tumorigenesis factor in KIRC.

Although the differences in CBX8 expression between PAAD and normal tissues was not observed in the TCGA project (tumor:178 cases, normal:4 cases) (Figure 1A), we found significant differences by combing the TCGA normal and GTEx (tumor:179 cases, normal:171 cases, *P*-value < 0.001) (Supplementary Figure 3B). When we performed statistical analysis on the expression of CBX8 and PAAD prognosis, we observed that high expression of CBX8 indicates better OS and DFS for patients in GEPIA, which is different from other tumors. From the Cox regression analysis, we can also see this correlation (Cox coefficient: −0.341, *P*-value: 1.70E-03) (Supplementary Table 3). The Kaplan-Meier data analysis results verified this conclusion, which indicates that high expression of CBX8 is closely related to better OS and RFS (Nagy et al., 2021). The conclusion was not be changed in different subgroups (Supplementary Table 1). This prognostic connection that is different from other tumors has no related reports for the time being, which points to a potential direction for future research and is worthy of further analysis.

We combined information on CBX8-binding components and CBX8 expression-related genes across all tumors to perform a series of enrichment analyses. The results show that the main biological processes of CBX8 include histone modification (histone ubiquitination, histone H2A monoubiquitination and others) and covalent chromatin modification, which have been reported to take part in gene transcription and repair of DNA damage (Zhou et al., 2009; Uckelmann and Sixma, 2017). According to the existing inclusion criteria, the main function of CBX8 is to regulate the biological activities of cells by inhibiting the transcription of some genes and repairing damaged DNA (Bardos et al., 2000; Lee et al., 2013). The above biological processes may have potential effects on cancer etiology or pathogenesis.

As an important part of the tumorigenesis and development process, the tumor immune microenvironment (TME) has a great impact on the biological behaviors of tumors (Gajewski et al., 2013). Immunotherapy strategies are gradually emerging based on the interaction between various immune cells that make up the TME and tumor cells (Pitt et al., 2016; Lei et al., 2020). We used multiple methods to evaluate the association between CBX8 levels and tumor-associated immune cells. For cancer-associated fibroblasts, CD8 + T cells, or B cells, the study indicated that CBX8 expression was correlated with the level of immune infiltration of different tumor-associated immune cells. CBX8 expression level has positive association with immune-cells infiltration level for some cancer, but contrary with others. It is similar with the survival analysis. CBX8 may affect the survival status of patients by changing the immune-cells infiltration in tumor microenvironment, which requires further research.

Through the CPTAC dataset, we explored expression differences in CBX8 phosphoprotein at different phosphorylated sites between five kinds of tumors (clear cell renal cell carcinoma, breast cancer, ovarian cancer, lung adenocarcinoma, and uterine corpus endometrial carcinoma) and the related normal tissues. The results showed higher expression of CBX8 phosphoprotein at the S191, S195, S196, S256, S264, and S311 loci in tumor tissues than in normal tissues. Although related articles have explored the possible role of phosphorylation sites (such as S256) (Chen et al., 2009), further experiments are necessary to explore the relationship between CBX8 phosphorylation and tumorigenesis.

Overall, our first pancancer analysis showed a significant association between CBX8 expression and patient clinical prognosis, protein phosphorylation, gene mutation, and immune cell infiltration, which helps us understand the potential mechanism of CBX8 in tumorigenesis and its clinical prognostic value.

## Conclusion

The level of expression of CBX8 is closely related to tumor carcinogenesis and prognosis. In the future, more experimental verification is needed to explore the significance of CBX8 in tumorigenesis or clinical prognosis in some different cancers.

## Data Availability Statement

The original contributions presented in the study are included in the article/Supplementary Material, further inquiries can be directed to the corresponding author.

## Author Contributions

DS and LA: conceptualization, methodology, visualization, and writing – original draft. HY: resources and data curation. YX: resources. JL and WZ: methodology, and writing – original draft. HX: conceptualization, supervision, and writing – original draft. All authors contributed to the article and approved the submitted version.

## Conflict of Interest

The authors declare that the research was conducted in the absence of any commercial or financial relationships that could be construed as a potential conflict of interest.

## Publisher’s Note

All claims expressed in this article are solely those of the authors and do not necessarily represent those of their affiliated organizations, or those of the publisher, the editors and the reviewers. Any product that may be evaluated in this article, or claim that may be made by its manufacturer, is not guaranteed or endorsed by the publisher.
