# Supplementary material for: Chromobox Homolog 8 (CBX8) in Human Tumor Carcinogenesis and Prognosis: A Pancancer Analysis Using Multiple Databases
Source: Front Genet. 2021 Sep 9;12:745277. doi: 10.3389/fgene.2021.745277 (PMC8458824; doi:10.3389/fgene.2021.745277)
Supplement: Supplementary file 1 [file Data_Sheet_1.docx]

Supplementary materials

Table. S1: Prognostic data for CBX8 in LIHC, BRCA, PAAD, and KIRC. We used the Kaplan-Meier plotter tool to analyze the prognostic data among different subgroups of four tumors: LIHC, BRCA, PAAD, and KIRC. Including OS, RFS, PFS, DSS, DMFS, and PPS. ^*^ *P* <0 .05; ^**^ *P* < 0.01; ^***^ *P* < 0.001. Symbol ^a^ indicates that CBX8 has a positive correlation with the prognostic data about the tumor, and symbol ^b^ indicates a negative correlation.

| Subtype | | OS | RFS | PFS | DSS | DMFS | PPS |
| --- | --- | --- | --- | --- | --- | --- | --- |
| LIHC | Alcohol consumption | 0.0011^**a^ | 0.001^**a^ | 0.0019^**a^ | 0.011^*a^ | / | / |
|  | No alcohol | 0.054 | 0.15 | 0.096 | 0.19 | / | / |
|  | Hepatitis virus | 0.0069^**a^ | 0.089 | 0.037^*a^ | 0.19 | / | / |
|  | No hepatitis virus | 0.012^*a^ | 0.16 | 0.31 | 0.065 | / | / |
| BRCA | P53 mutated | 0.041^*b^ | 0.2 | / | / | 0.029^*b^ | 0.082 |
|  | P53 wild | 0.23 | 0.0037^**a^ | / | / | 0.11 | 0.02^*b^ |
| PAAD | Female | 0.018^*b^ | 0.0097^**b^ |  |  |  |  |
|  | Male | 4.40E-06^***b^ | 0.00043^***b^ | / | / | / | / |
|  | White | 2.70E-05^***b^ | 0.0019^**b^ | / | / | / | / |
|  | Asian | / | / | / | / | / | / |
|  | Black/African American | / | / | / | / | / | / |
| KIRC | Female | 0.00043^***a^ | 0.017^*b^ | / | / | / | / |
|  | Male | 8.70E-10^***a^ | 0.011^*a^ | / | / | / | / |
|  | White | 9.80E-10^***a^ | 0.1 | / | / | / | / |
|  | Asian | / | / | / | / | / | / |
|  | Black/African American | 0.011^*a^ | 0.13 | / | / | / | / |

Table. S2: The *P*-value between genetic alteration of CBX8 and clinical prognosis in different types of cancer (except Rectum adenocarcinoma).

| Cancer Type | OS | DSS | DFS | PFS |
| --- | --- | --- | --- | --- |
| Adrenocortical Carcinoma | 1.39E-03 | 0.161 | 0.442 | 0.535 |
| Bladder Urothelial Carcinoma | 0.267 | 0.842 | 0.894 | 0.985 |
| Breast Invasive Carcinoma | 0.417 | 0.658 | 0.841 | 0.872 |
| Brain Lower Grade Glioma | 0.266 | 0.726 | 0.793 | 0.98 |
| Glioblastoma Multiforme | 0.141 | 0.553 | / | 0.233 |
| Cervical Squamous Cell Carcinoma | 0.346 | 0.457 | 0.674 | 0.702 |
| [Esophageal Adenocarcinoma](https://www.cbioportal.org/study?id=esca_tcga_pan_can_atlas_2018) | 6.09E-04 | 0.349 | 0.552 | 0.863 |
| [Stomach Adenocarcinoma](https://www.cbioportal.org/study?id=stad_tcga_pan_can_atlas_2018) | 0.685 | 0.766 | 0.885 | 0.89 |
| Head and Neck Squamous Cell Carcinoma | 0.489 | / | 0.188 | 0.264 |
| Kidney Renal Clear Cell Carcinoma | 0.593 | 0.693 | 0.18 | 0.0601 |
| [Liver Hepatocellular Carcinoma](https://www.cbioportal.org/study?id=lihc_tcga_pan_can_atlas_2018) | 0.95 | 0.579 | 0.934 | 0.791 |
| [Lung Adenocarcinoma](https://www.cbioportal.org/study?id=luad_tcga_pan_can_atlas_2018) | 0.719 | 0.763 | 0.866 | 0.915 |
| [Ovarian Serous Cystadenocarcinoma](https://www.cbioportal.org/study?id=ov_tcga_pan_can_atlas_2018) | 0.677 | 0.597 | 0.439 | 0.518 |
| [Pancreatic Adenocarcinoma](https://www.cbioportal.org/study?id=paad_tcga_pan_can_atlas_2018) | 0.241 | 0.153 | / | 0.53 |
| [Mesothelioma](https://www.cbioportal.org/study?id=meso_tcga_pan_can_atlas_2018) | 0.0291 | 0.0635 | / | 0.115 |
| [Prostate Adenocarcinoma](https://www.cbioportal.org/study?id=prad_tcga_pan_can_atlas_2018) | 0.885 | 0.929 | 0.697 | 0.0955 |
| [Skin Cutaneous Melanoma](https://www.cbioportal.org/study?id=skcm_tcga_pan_can_atlas_2018) | 0.836 | 0.805 | / | 0.734 |
| [Uterine Carcinosarcoma](https://www.cbioportal.org/study?id=ucs_tcga_pan_can_atlas_2018) | 0.34 | 0.352 | 0.13 | 0.978 |
| [Uterine Corpus Endometrial Carcinoma](https://www.cbioportal.org/study?id=ucec_tcga_pan_can_atlas_2018) | 0.173 | 0.354 | 0.367 | 0.359 |
| [Thyroid Carcinoma](https://www.cbioportal.org/study?id=thca_tcga_pan_can_atlas_2018) | 0.902 | 0.932 | 3.40E-04 | 0.0268 |
| [Sarcoma](https://www.cbioportal.org/study?id=sarc_tcga_pan_can_atlas_2018) | 0.884 | 0.652 | 0.445 | 0.228 |

Table. S3: Cox regression results for CBX8 in different tumors via the Oncolnc database.

| Cancer | Cox Coefficient | *P-Value* |
| --- | --- | --- |
| BLCA | -0.075 | 3.20E-01 |
| BRCA | -0.111 | 2.20E-01 |
| CESC | 0.127 | 3.40E-01 |
| COAD | 0.034 | 7.30E-01 |
| ESCA | 0.069 | 6.20E-01 |
| GBM | -0.15 | 1.10E-01 |
| HNSC | 0.014 | 8.40E-01 |
| KIRC | 0.355 | 8.60E-06 |
| KIRP | 0.096 | 5.10E-01 |
| LAML | 0.067 | 5.60E-01 |
| LGG | 0.194 | 5.30E-02 |
| LIHC | 0.21 | 2.50E-02 |
| LUAD | -0.078 | 3.30E-01 |
| LUSC | -0.006 | 9.20E-01 |
| OV | -0.214 | 3.80E-03 |
| PAAD | -0.341 | 1.70E-03 |
| READ | -0.128 | 5.20E-01 |
| SARC | 0.014 | 8.90E-01 |
| SKCM | 0.095 | 1.70E-01 |
| STAD | -0.158 | 6.40E-02 |
| UCEC | -0.061 | 5.50E-01 |

Figure S1. Phylogenetic tree of CBX8. The phylogenetic tree of CBX8 in different species was generated by the constraint-based multiple alignment tool from NCBI.

Figure S2. RNA expression of CBX8 in different tissues, single cell types, and blood cells in a normal physiological state. (A) The expression of CBX8 in different tissues using the consensus datasets from HPA, GTEx, and FANTOM5. (B) The comprehensive expression of CBX8 in different single cell types based on colon, eye, heart muscle, kidney, liver, lung, pancreas, placenta, prostate, rectum, skin, small intestine, and testis tissues. (C) The expression of CBX8 in different blood cells using the consensus datasets of the HPA scaled dataset, Monaco scaled dataset, and Schmiedel dataset.

Figure S3. Expression of the CBX8 gene in different tumors. CBX8 expression differences between ACC, KIRC, PAAD, and SARC in the TCGA project and the corresponding normal tissues in the GTEx databases, * *P*<0.01.

Figure S4. Pooled analysis of CBX8 expression differences between normal and tumor tissues based on the Oncomine database. (A) Colorectal cancer. (B) Leukemia. (C) Liver cancer. (D) Lung cancer. (E) Sarcoma.

Figure S5. Expression of the CBX8 gene in different pathological stages of various tumors. All enrolled cancers included UCEC, SKCM, ACC, LUAD, COAD, KIRP, KIRC, STAD, READ, UCS, ESCA, LIHC, LUSC, DLBC, BRCA, BLCA, PAAD, NHSC, CHOL and TGCT.

Figure S6. Correlation between CBX8 gene expression and prognosis in different cancers. The relationship between CBX8 expression and prognosis in the four types of cancers was explored via the Kaplan-Meier plotter, including OS, DMFS, RFS, PFS, PPS, and DSS. (A) KIRC. (B) LIHC. (C) Breast cancer. (D) PAAD.

Figure S7. Correlation between CBX8 expression and immune infiltration of different immune cells. Different algorithms were used to evaluate the relationship between CBX8 expression and immune infiltration of different immune cells (CD8+ T-cells and B cells) in all types of TCGA cancer. (A) CD8+ T-cells. (B) B cells.

Figure S8. GO biological processes and GO cellular component analysis in tumors based on the CBX8-binding and interacting genes, which are visualized via cnetplots. (A) Biological process. (B) Cellular component.
