# Supplementary figures and images for: Chromobox Homolog 8 (CBX8) in Human Tumor Carcinogenesis and Prognosis: A Pancancer Analysis Using Multiple Databases
Source: Front Genet. 2021 Sep 9;12:745277. doi: 10.3389/fgene.2021.745277 (PMC8458824; doi:10.3389/fgene.2021.745277)

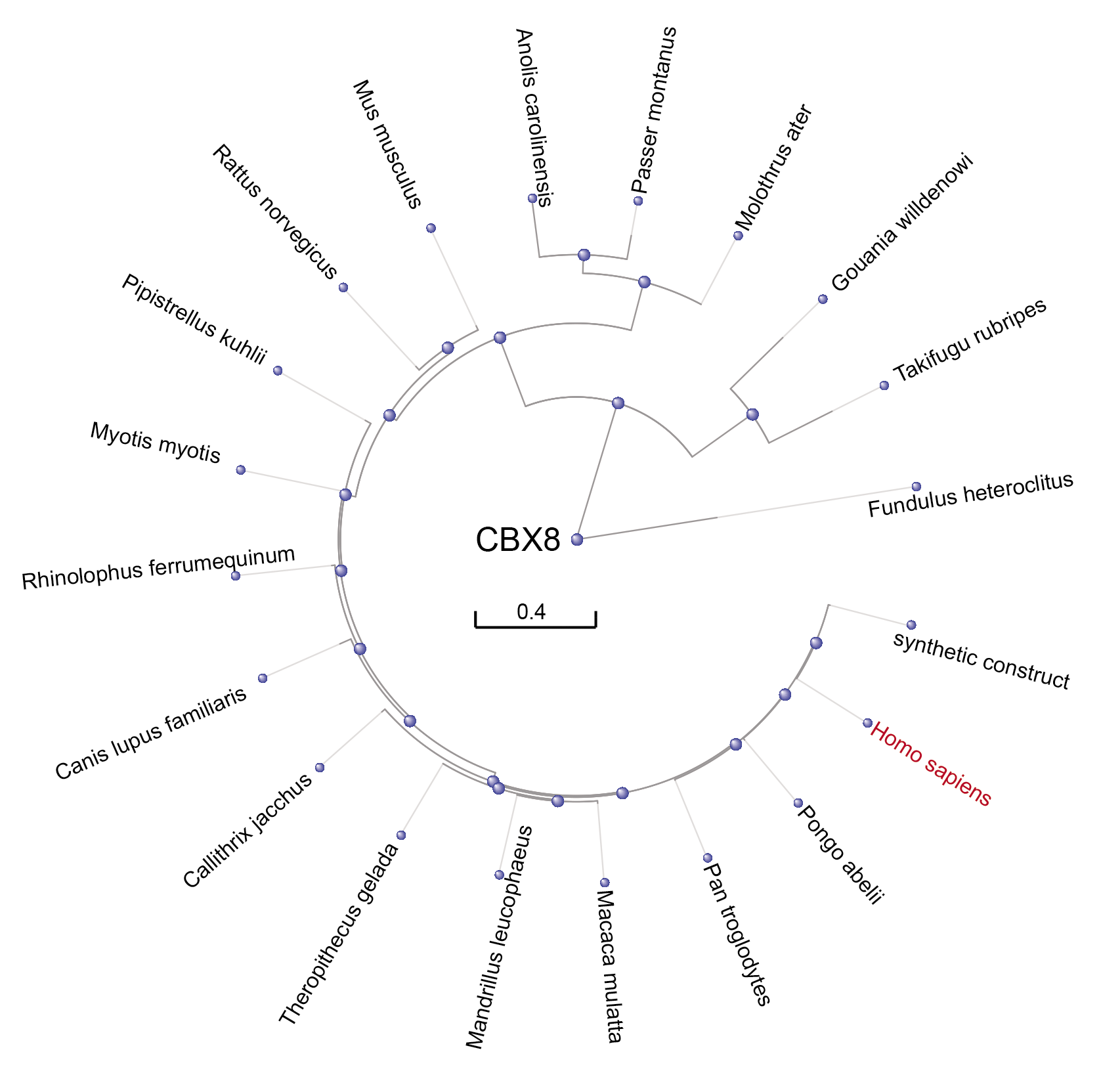

Supplement: Supplementary file 2 [file Image_1.TIF]

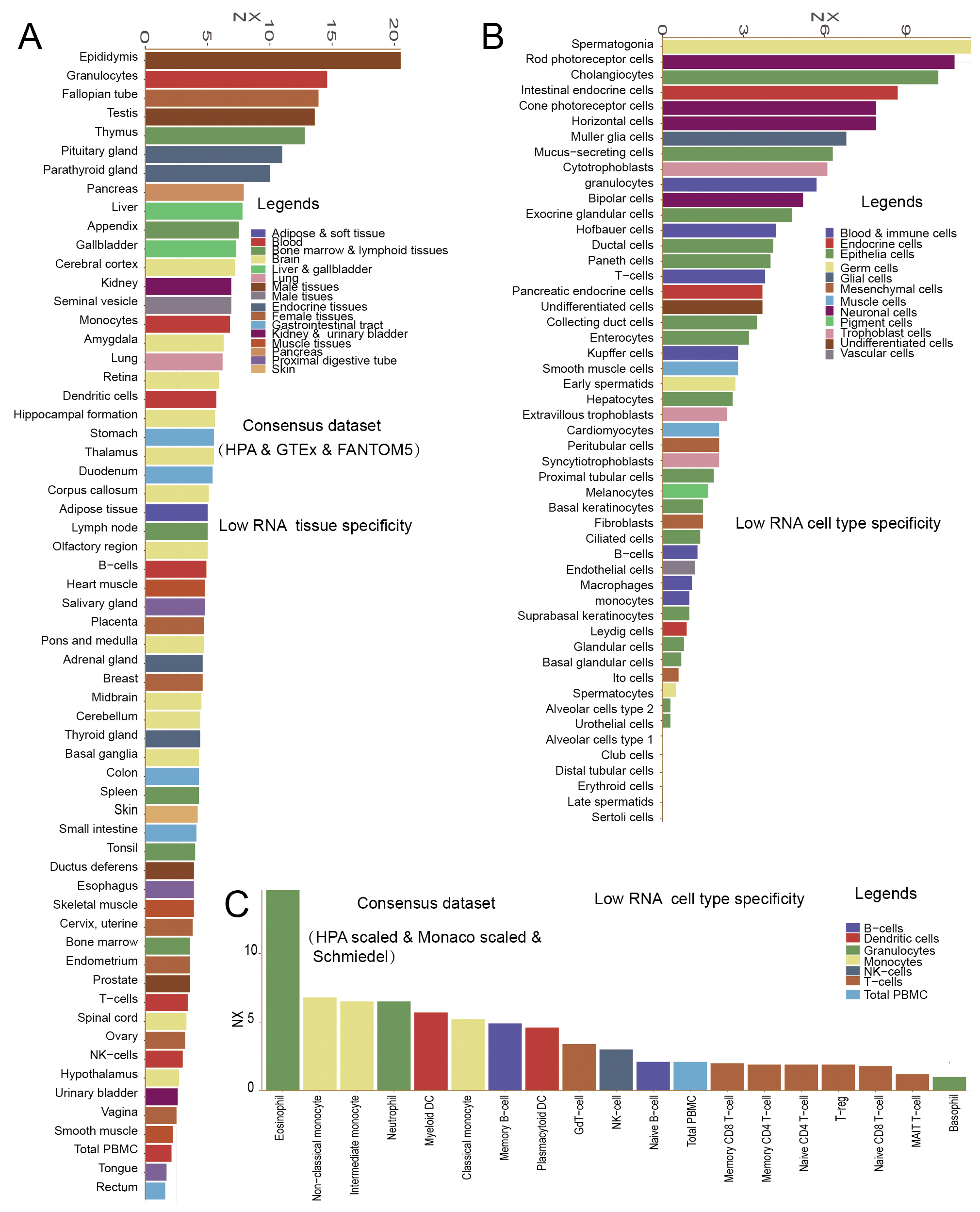

Supplement: Supplementary file 3 [file Image_2.TIF]

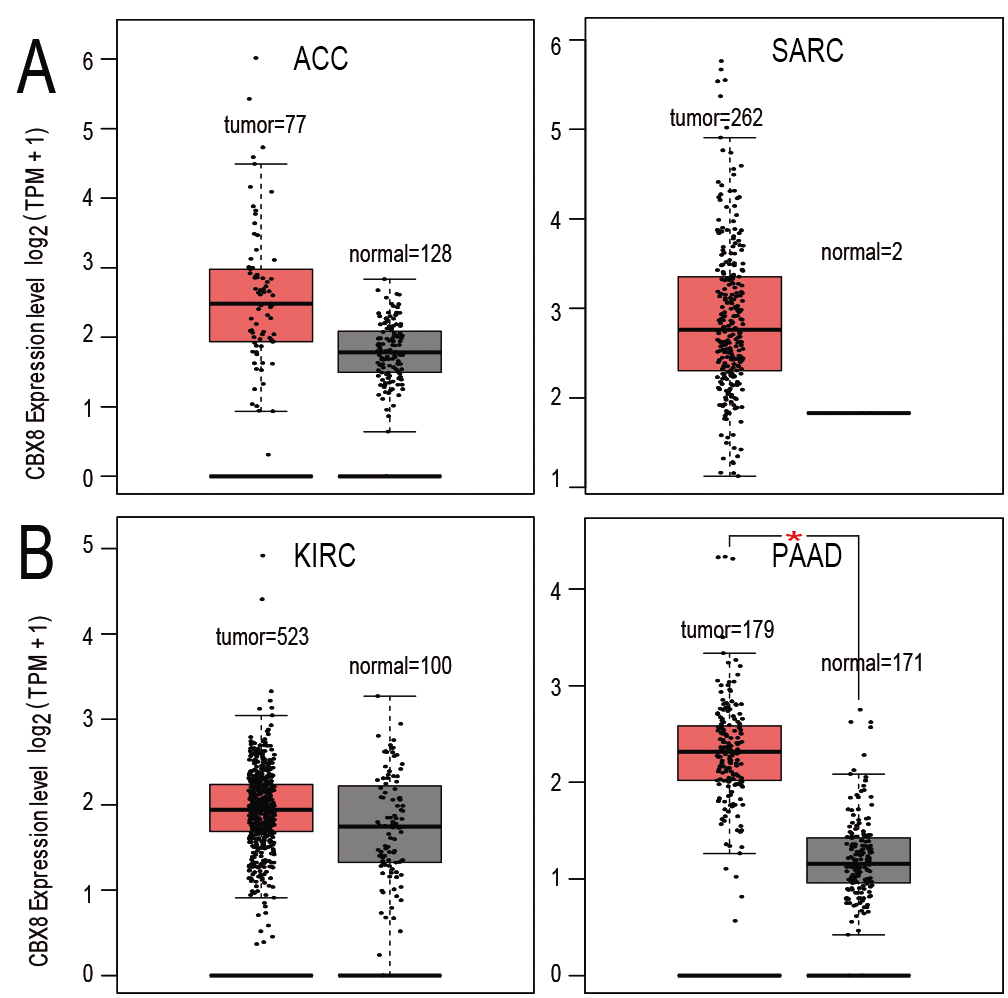

Supplement: Supplementary file 4 [file Image_3.TIF]

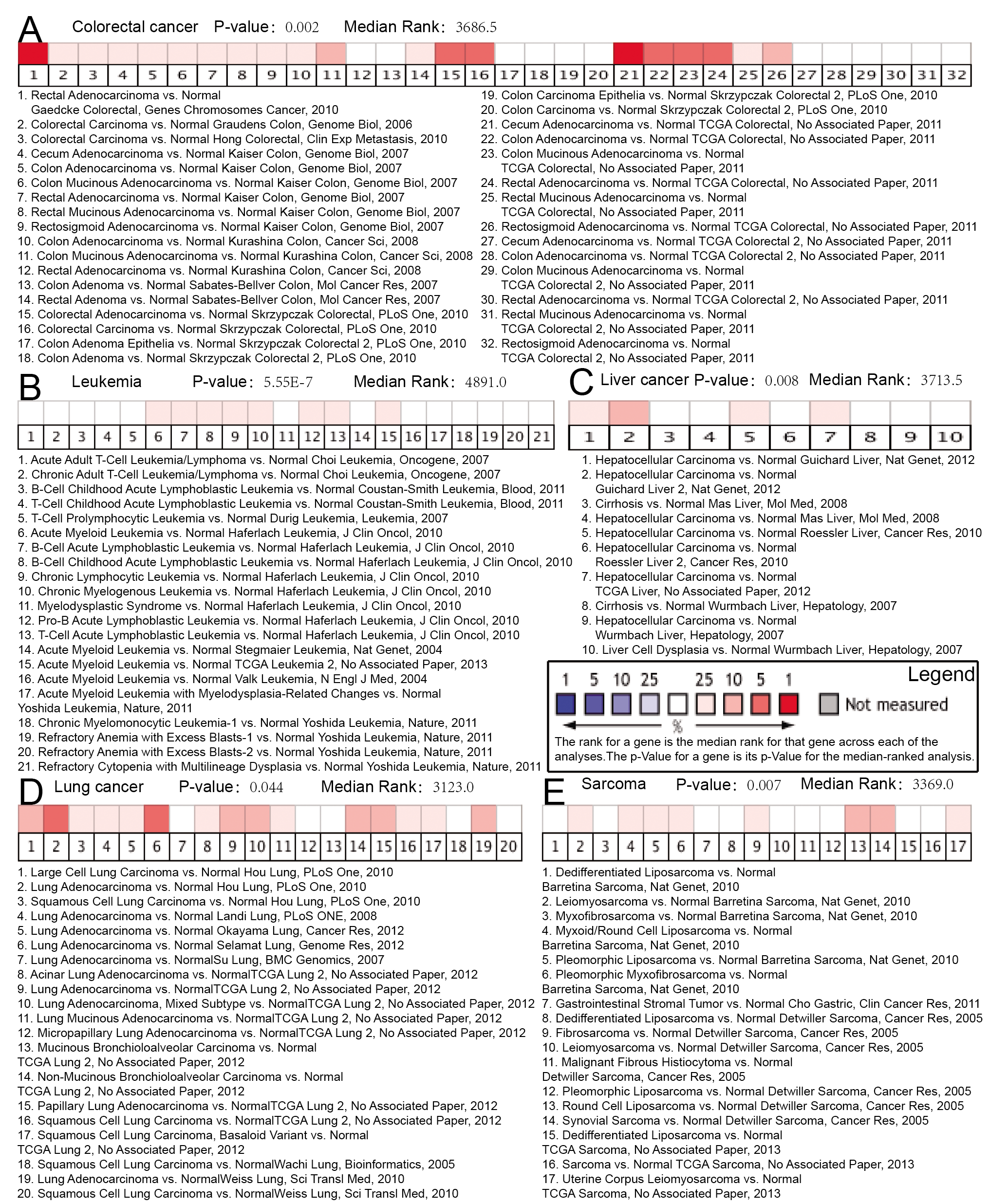

Supplement: Supplementary file 5 [file Image_4.TIF]

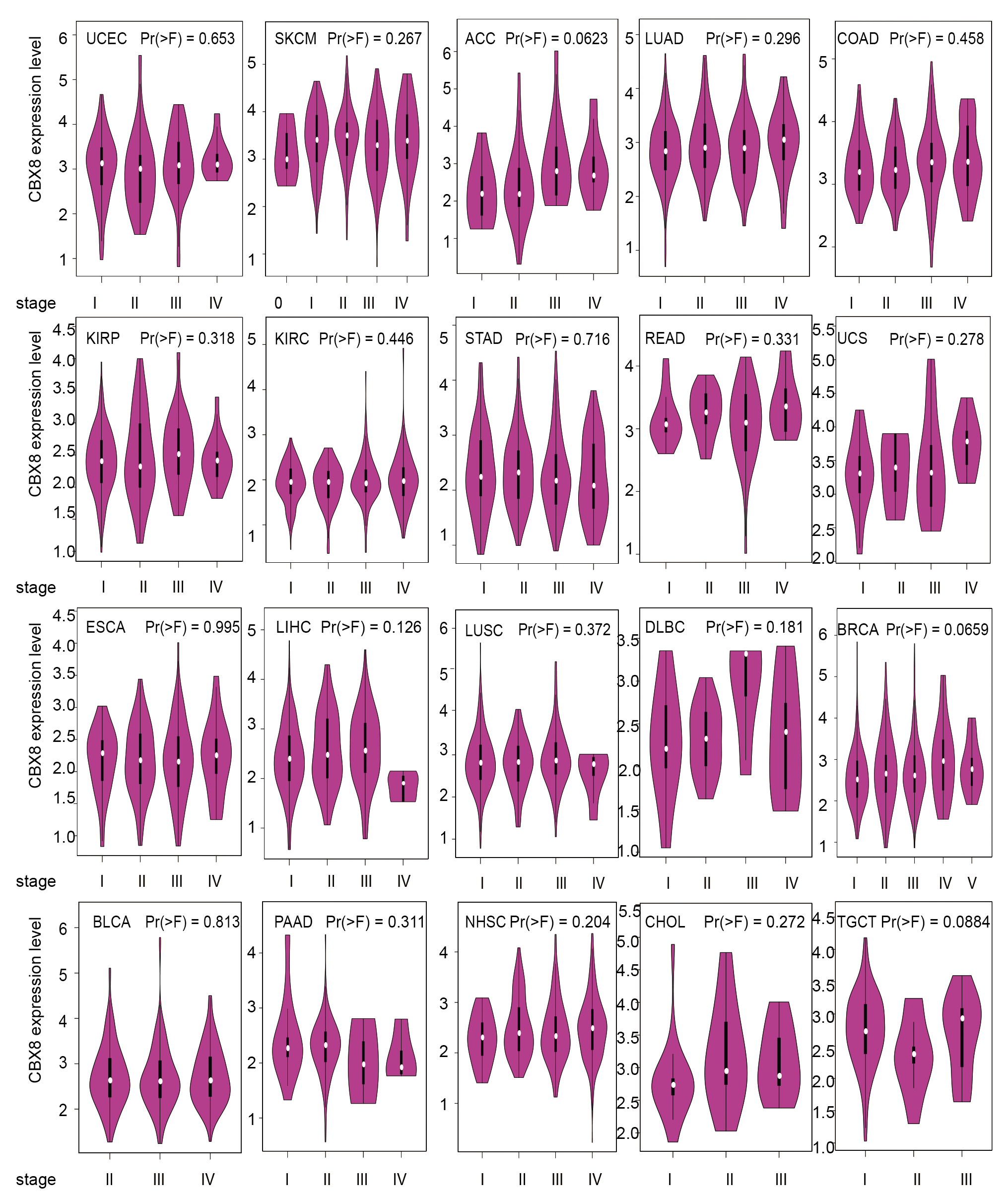

Supplement: Supplementary file 6 [file Image_5.TIF]

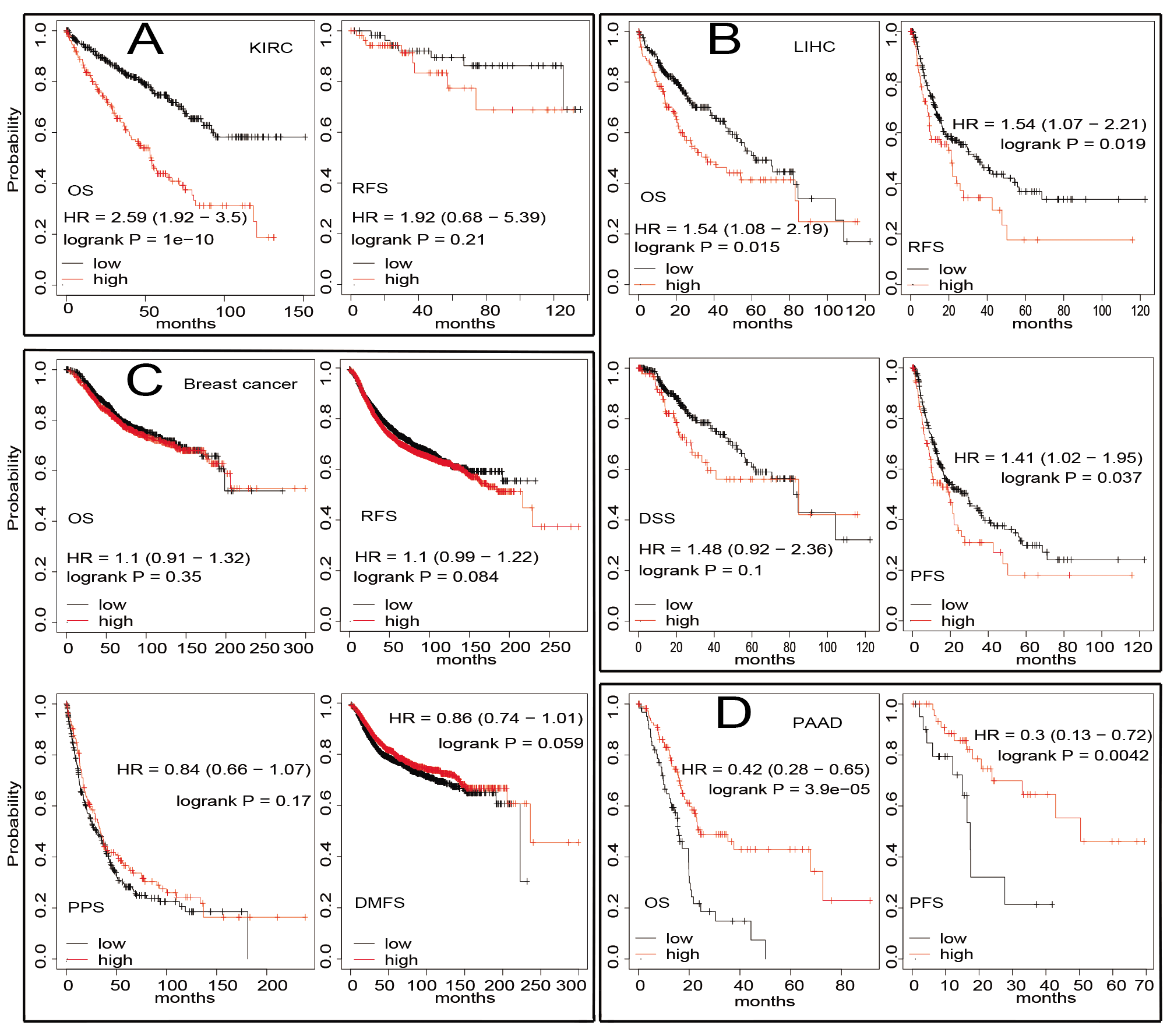

Supplement: Supplementary file 7 [file Image_6.TIF]

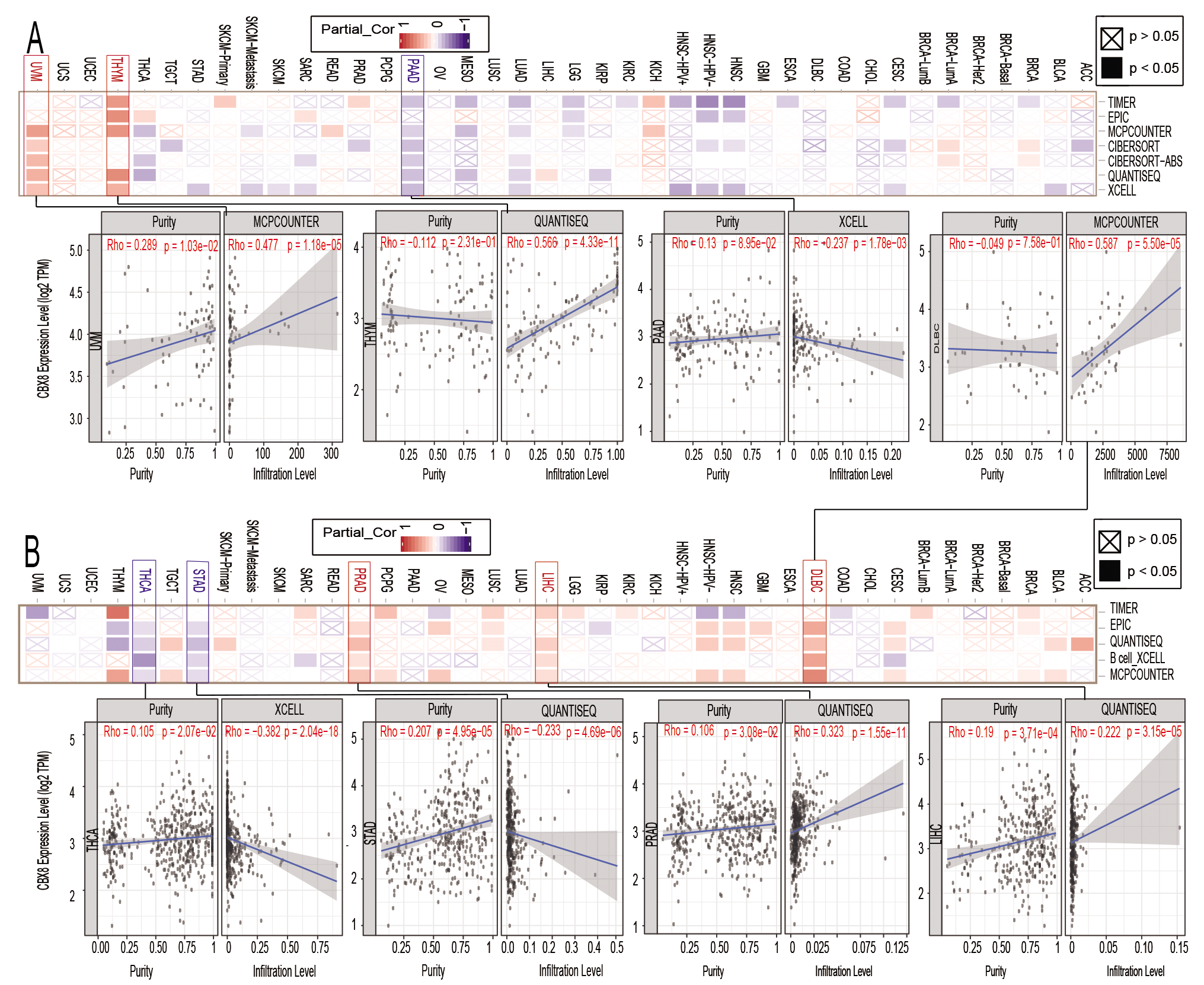

Supplement: Supplementary file 8 [file Image_7.TIF]

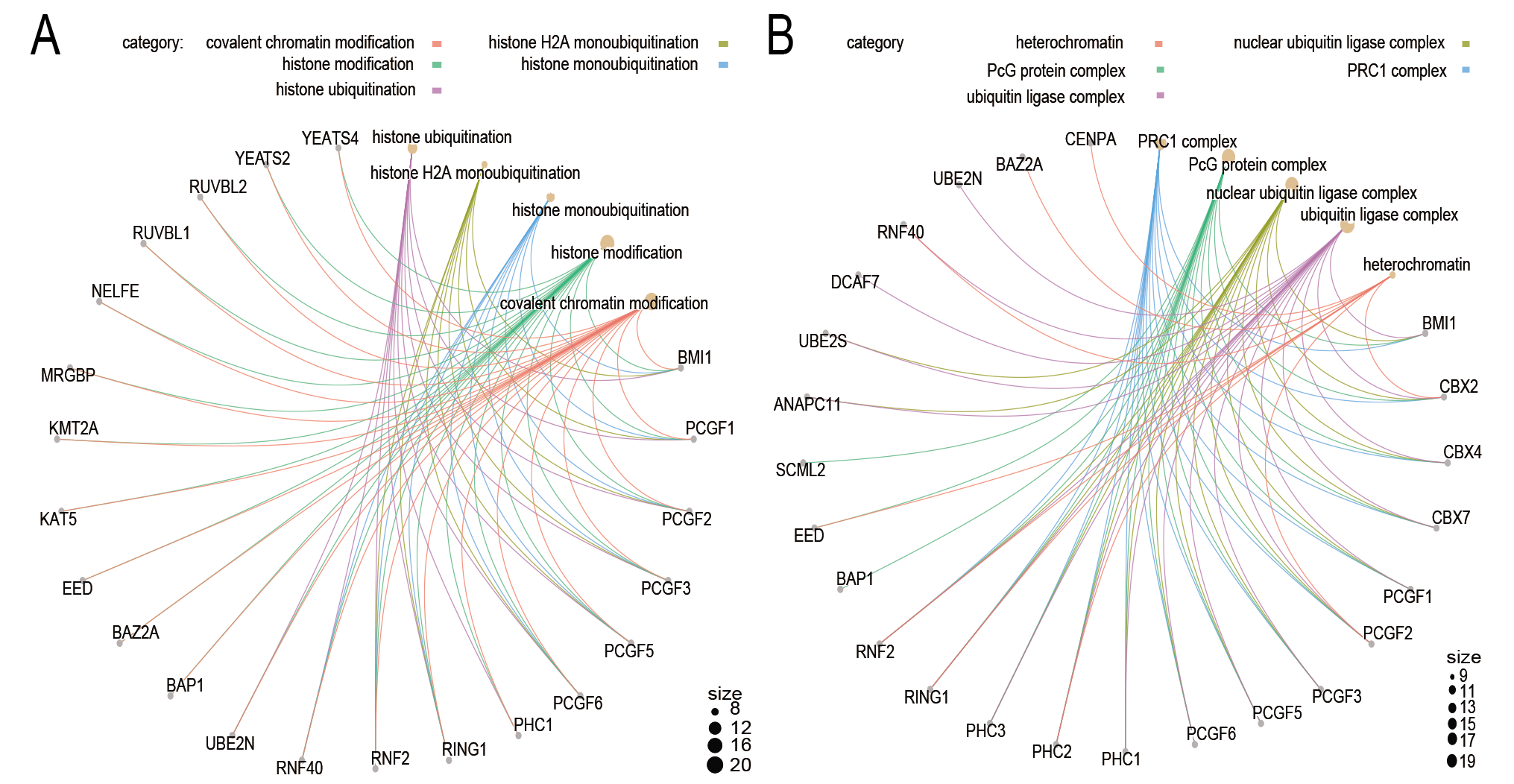

Supplement: Supplementary file 9 [file Image_8.TIF]
